# A rare case report: tricuspid valve prolapse due to spontaneous chordae rupture in a congenitally corrected transposition of the great arteries patient

**DOI:** 10.1186/s13019-020-01193-0

**Published:** 2020-06-29

**Authors:** Wan Yu Hu, Bo Wen Zhao, Shi Yan Li, Bei Wang

**Affiliations:** Department of Diagnostic Ultrasound & Echocardiography, Sir Run Run Shaw Hospital, Zhejiang University School of Medicine, No.3 East Qingchun Road, Hangzhou, 310016 People’s Republic of China

**Keywords:** Case report, Congenitally corrected transposition of the great arteries, Spontaneous chordae rupture, Tricuspid valve prolapse, Real-time three dimentional transesophageal echocardiography

## Abstract

**Background:**

Congenitally corrected transposition of great arteries (CCTGA) is caused by atrioventricular and ventriculoarterial discordance. Cases of CCTGA with spontaneous chordae rupture of tricuspid valve have not been reported before.

**Case presentation:**

Here we diagnosed a 38-year-old man, who was found CCTGA 14 years ago, as spontaneous chordae rupture by real-time three dimentional transesophageal echocardiography (RT-3D-TEE). The present case is the first report to describe a CCTGA patient combine with spontaneous chordae rupture in tricuspid valve. After tricuspid valve replacement, the patient was uneventful after 6 years’ follow-up.

**Conclusion:**

We reported a rare case with spontaneous chordae rupture of tricuspid valve in a CCTGA patient and explored its etiology here. RT-3D-TEE is an important supplement to 2-dimentional transthoracic echocardiography and can provide more accurate detections in tricuspid valve diseases in CCTGA.

## Introduction

Congenitally corrected transposition of the great arteries (CCTGA) only occurs in 0.5% of patients exhibiting congenital heart defects. It is a slight male predominance. The right sided morphologic left ventricle functions as the pulmonary ventricle, whereas the left sided morphologic right ventricle functions as the systemic ventricle. The systemic atrioventricular valve is morphologically tricuspid valve (TV) [1]. To the best of our knowledge, no case of spontaneous chordae rupture of TV in CCTGA patient was reported before.

## Case presentation

A 38-year-old male was referred to our hospital because of progressive exertional dyspnea and fatigue of 1 week. He was diagnosed as CCTGA in a physical examination without any symptoms 14 years ago. The heart ultrasound examination revealed CCTGA without any other anomalies, such as ventricular septal defect, pulmonary stenosis, atrial septal defect, etc. TV regurgitation was mild and ejection fraction (EF) of systemic ventricle was 67% at that time. He denied prior history of hypertension, coronary heart disease and diabetes. The initial vital signs were normal (heart rate was 93 beats/min, blood pressure was 110/70 mmHg, breath rate was 16/min with an oxygen saturation 99%, and temperature was 36.8 °C). Physical examination revealed a grade 4/6 holosystolic murmur at the left sternal border, accompanied by a thrill. Mild lower extremity edema was present. Heart function in New York Heart Association (NYHA) was Class III.

**Fig. 1:**
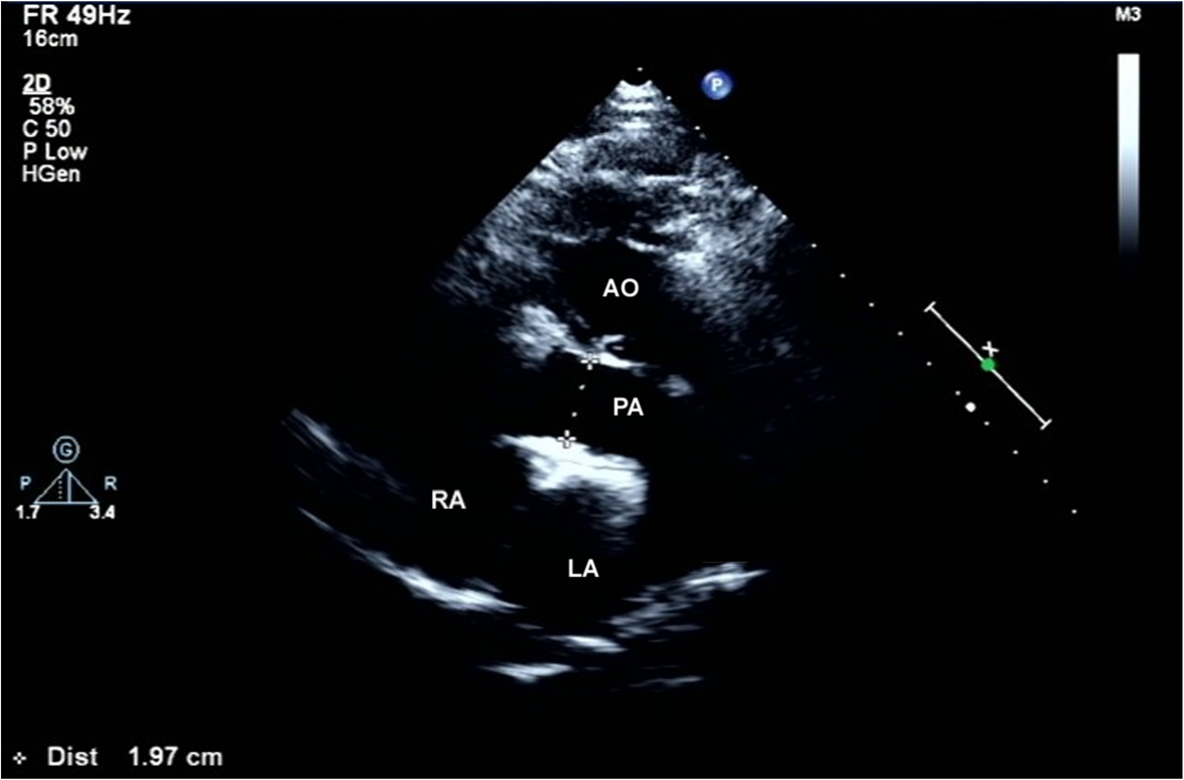
Two dimensionally parasternal long-axis view showing the aorta lied in anterior left side of pulmonary artery and connected to morphological right ventricle (functional left ventricle). AO = aorta; PA = pulmonary artery; LA = left atrial; RA = right atrial

**Fig. 2:**
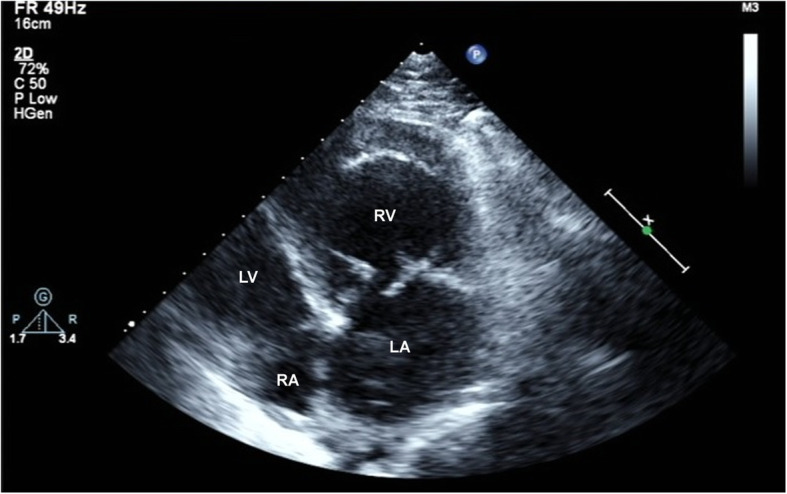
Apical four-chamber view showed the valve in morphological left ventricle was inferior to the valve in the right ventricle. Anterior leaflet of tricuspid valve receded to left atrium and left a large gap with other leaflets in systole period. LA = left atrial; RA = right atrial; RV = right ventricle; LV = left ventricle

**Fig. 3:**
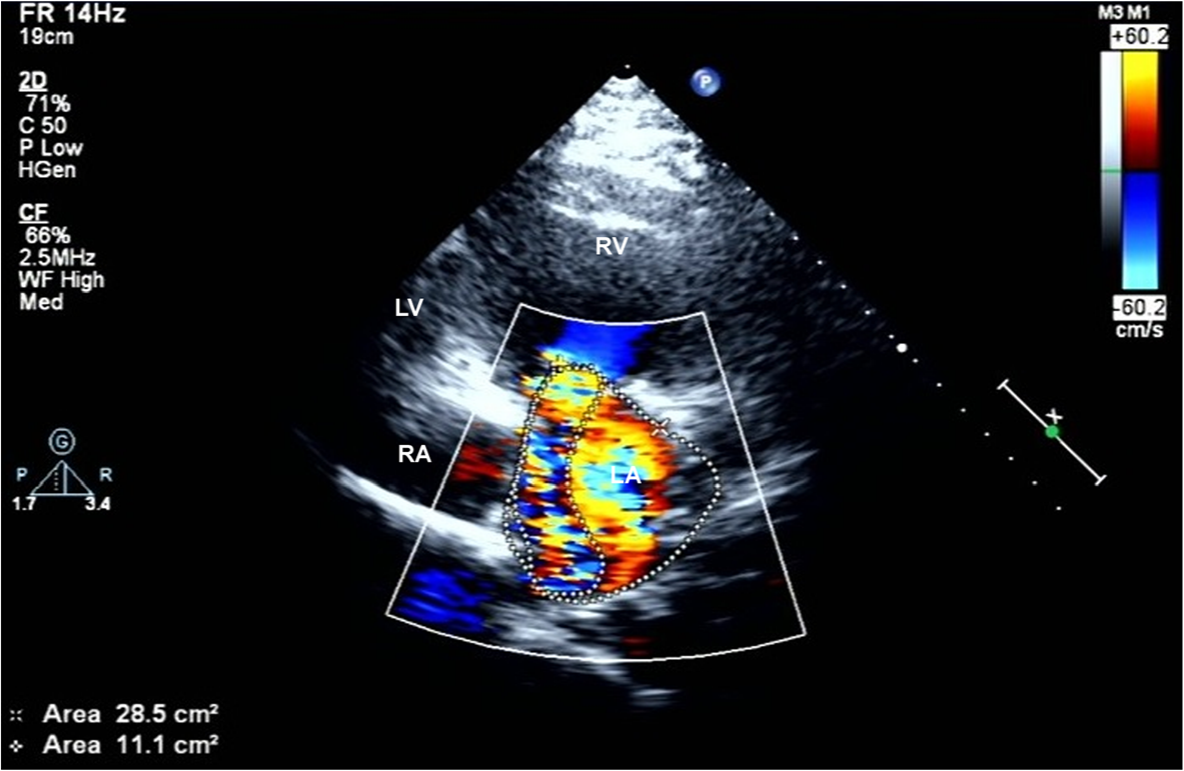
Severe tricuspid valve regurgitation jet was visible in Color Doppler mode. LA = left atrial; RA = right atrial; RV = right ventricle; LV = left ventricle

Laboratory evaluation was normal. White blood cell count 4700/mL (normal 4000–10,000/mL), C-reactive protein level was 0.09 mg/dL (0.0–0.5 mg/dL), and erythrocyte sedimentation rate was 5 mm/h (normal 0–15 mm/h). Electrocardiogram indicated sinus rhythm. A 2-dimentional transthoracic echocardiography (2D-TTE) examination was performed on IE33 machine (Philips Healthcare, Amsterdam, NL). The S5–1 sector array probe was used and its frequency range was 1–5 MHz. TTE revealed liver lay in the right side, spleen and stomach lay in the left side. A series of images were obtained from different windows including parasternal long-axis view (Fig. 1), parasternal short-axis view and apical view (Fig. 2). Atrioventricular and ventricular arterial were discordant. The morphological right ventricle, connecting to the left atrium and aorta, was placed leftward and functioned as systemic ventricle. Similarly, the morphological left ventricle, connecting to the right atrium and pulmonary artery, was placed rightward and functioned as a pulmonary ventricle. The anterior leaflet of TV was prolapsed and could not be aligned with the posterior valve, leaving a large gap (Fig. 2). Color Doppler showed severe TV regurgitation flowed into the dilated left atrium (Fig. 3). System ventricle was enlarged and its end-diastolic diameter was 58 mm, EF of systemic ventricle was 50%.

**Fig. 4:**
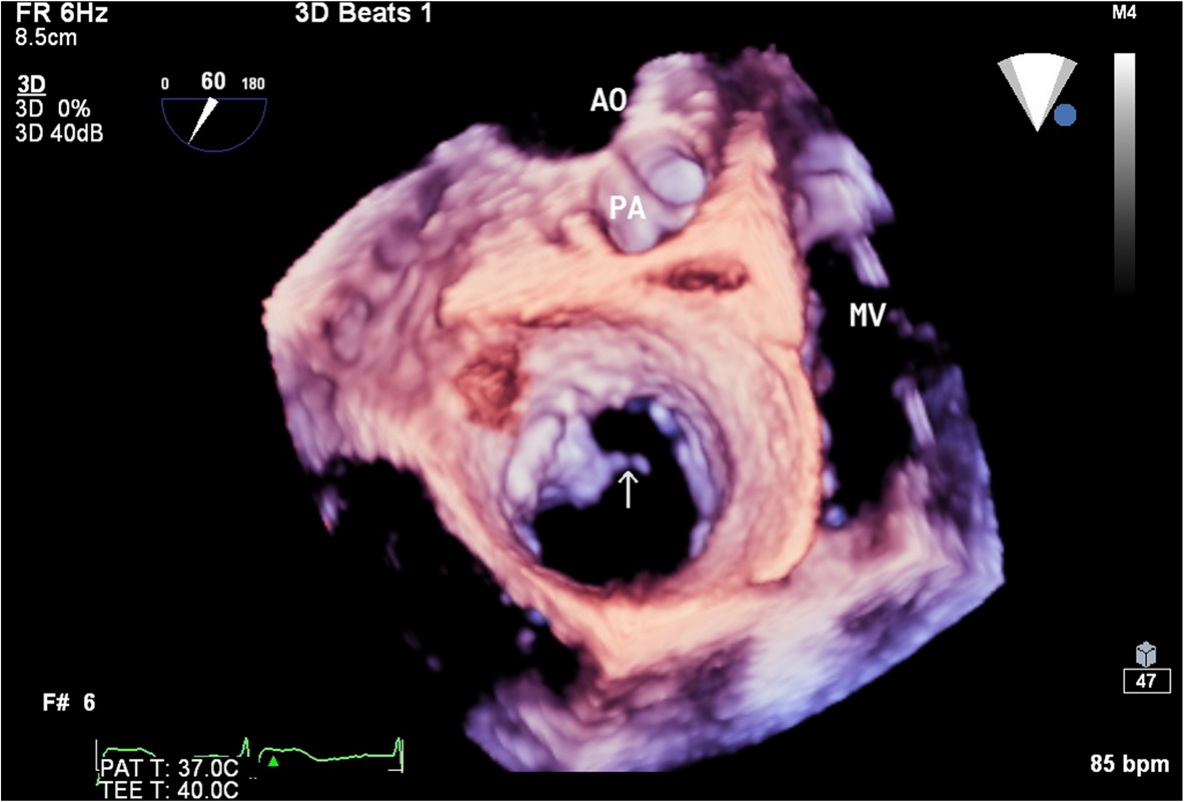
RT-3D-TEE showed rupture chordae (arrow) attached the anterior leaflet of tricuspid valve in diastole period. AO = aorta; PA = pulmonary artery; MV = mitral valve

However, with 2D-TTE, the reason of TV prolapse was unclear and the image was poor due to the shadow of ribs. Real-time three-dimentional transesophageal echocardiography (RT-3D-TEE) was performed to further define the anatomy of the TV. The probe used was X7-2t sector array probe and its frequency range was 2–7 MHz. We Obtained images of TV from either the 0° to 30° mid-esophageal view, four chamber view tilting to place the valve in the center or the 40° trans-gastric view with ante-flexion. 3D images of TV were acquired by using a narrow-angle, single-beat mode. After cropped 3D volume, we obtained real-time 3D imaging of TV. A short rod-like structure was seen on the left atrial side of TV. It swung back and forth with the flow of blood, causing a wide gap in the three valves of TV in systolic period (Fig. 4. The crime culprit of anterior leaflet prolapse was detected by RT-3D-TEE. It was rupture of one main chordae.

**Fig. 5:**
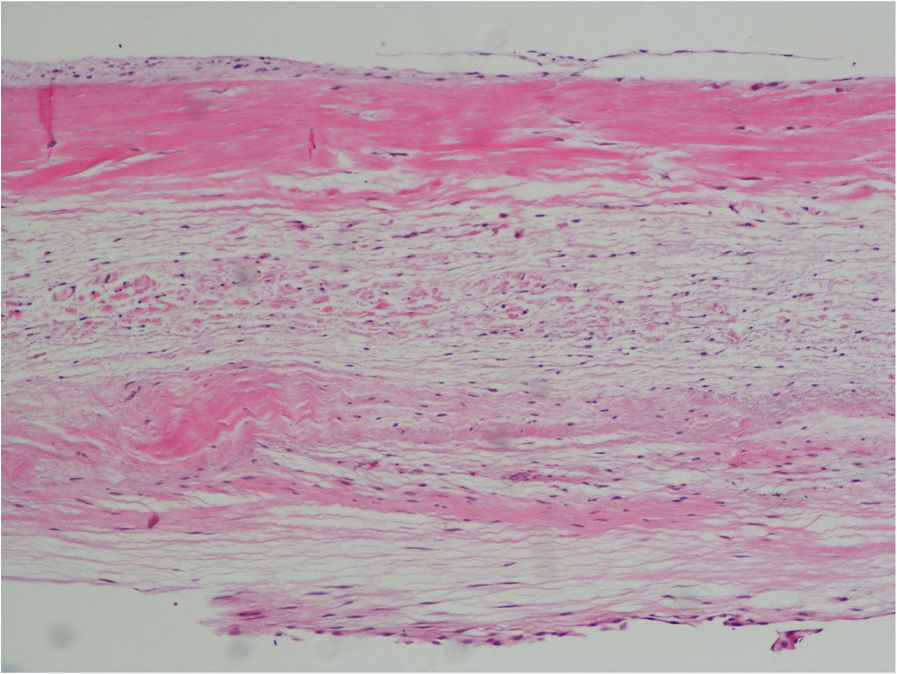
Microscopic appearance. TV was normal tissue without infective endocarditis and other disease (H&E, × 200)

The patient was diagnosed as CCTGA, chordae rupture of TV and severe TV regurgitation. Operation needed to be performed because of progressive exertional dyspnea and fatigue. Because TV repair was difficult in TV prolapse with chordae rupture and late results of valvuloplasty were poor in CCTGA, [2] we thought TV replacement was more suitable for this patient and then performed TV replacement surgery. Considering the patient’s young age and the size of heart, we chose a 27 mm Carbomedics bileaflet mechanical prosthesis. The surgery confirmed 3D TEE findings. Intra-operation showed the TV was intact, no perforation or tear was found, a rupture of chordae was observed in the anterior valve, the broken end was attached to the tip of the anterior valve, and no papillary muscle injury was observed in the three muscles. Visual inspection and histologic examination was normal and denied infective endocarditis (Fig. 5). Spontaneous chordae rupture was deemed to the etiology of TV prolapse after excluded other reasons.

The signs and symptoms of dyspnea were abated 3 months after surgery. Electrocardiogram revealed sinus rhythm. No regurgitation of the mechanical prosthesis was found by 2D-TTE. System ventricle end-diastolic diameter was 53 mm, the EF was 60% and inflow of TV was normal. Heart function was NYHA Class I. At 6 years’ follow up, the patient was uneventful without any complications. TTE revealed no TV regurgitation at last follow up (1 month ago). EF of systemic ventricle was 57%. System ventricle end-diastolic diameter was 51 mm. Inflow of TV was normal. Maximal velocity was 1.4 m/s, peak pressure gradient was 8 mmHg, mean pressure gradient was 3 mmHg, velocity time integral was 44.6 cm, pressure half time was 113 ms. Sinus rhythm was reported by electrocardiogram.

## Discussion

CCTGA is a rare congenital disease with a prevalence of 0.03 per 1000 live births. Patients with CCTGA may coexisted other anomalies, such as pulmonic stenosis, ventricular septal defect, atrioventricular block, atrial septal defect and TV abnormalities. Its presentation and prognosis is highly variable depending on these coexisting anomalies [3]. In up to 70% of cases, the TV is abnormal and may be inferiorly displaced [4]. However, spontaneous ruptured chordae of TV in CCTGA patient was not reported before. The etiology of spontaneous chordae rupture of TV is not clear up to now.

To further define the reason of chordae rupture, we asked previous history and found a period of 6 month moderate TV regurgitation. Therefore, we considered several factors may contribute to it in this patient. Firstly, in CCTGA, the TV is located within the systemic ventricle, resulting in ability of TV insufficiency. Because TV is more susceptible to regulation caused by pressure/volume overload. TV insufficiency in turns imposes an additional volume pressure on the overloaded morphologically right ventricular [5].. A vicious cycle was participated in valvular and ventricular dysfunctions. Secondly, right ventricle was high-volume and low-pressure system in normal heart. But in CCTGA, TV supplies the systemic circulation, which is a high-pressure system, through the aorta. Long-standing progression of TV regurgitation and increased volume pressure would be the etiology of isolated spontaneous rupture of TV apparatus in some patients. Thirdly, moderate TV regulation would reduce the output of heart and increased myocardial oxygen consumption, causing ischemia of myocardium and valve devices. Additionally, TV acted as mitral valve in CCTGA patient was more susceptible prolapsed due to histologic difference. This case indicated CCTGA patient with normal chordae and valve could also occur spontaneous chordae rupture. Histologic difference and hemodynamics mainly contributed to it. It is a rarely case of TV prolapse due to this reason. Our patient was uneventful during 6 years’ follow-up, indicating valve replacement surgery was efficient in these patients.

In a literature review, only two cases with TV prolapse in CCTGA were reported before. One was [5] an adult CCTGA patient with TV prolapse. His TV was congenital dysplastic and oriented down toward the septum abnormally but not Ebstein’s anomaly. Another was [6] a female CCTGA patient with steno-insufficiency of TV and prolapse of both TV and mitral valve. To the best of our knowledge, we are the first to report isolate TV prolapse caused by spontaneous chordae rupture in CCTGA patient. Our patient found spontaneous chordae rupture within a short time without any precipitating factor. His TV was normal and there were no other associated cardiac abnormalities in his heart.

2D-TTE cannot achieved simultaneous of the TV leaflets due to its non-planar geometry and complex geometry of the right ventricle. This patient could not be observed TV clearly due to rib shadow. RT-3D-TEE could overcome the disturbance of ribs, sternum, lung tissue and was not affected by emphysema. Moreover, RT-3D-TEE could show three-dimensional morphology of TV and the structural of complex atrioventricular valve device in atrial or ventricular side. RT-3D-TEE help surgeons to see the complicated multiform morphology directly and evaluate dynamics of valve regurgitation [6, 7]. Through the “en face view”, an obviously broken chordae tendineae was detected in 3D imaging. Chordae rupture was diagnosed easily. RT-3D-TEE detected the cause of TV prolapse, which was rupture of one main chordae. But 2D-TEE did not found it. Therefore, we speculated that RT-3D-TEE would be a crucial examination in diagnosing TV diseases and can provide more accurate and additional diagnoses for CCTGA patients than TTE.

## Conclusion

In summary, we diagnosed a CCTGA patient with chordae rupture with RT-3D-TEE here and indicated CCTGA patient with normal chordae and valve can also occur spontaneous chordae rupture. Valve replacement surgery was useful in patient by a 6 years’ follow up. Furthermore, we demonstrated RT-3D-TEE played an important role in diagnosing TV diseases and could provide additional and more accurate information than TTE in CCTGA patients.

## Data Availability

Not applicable.

## Abbreviations

CCTGA: Congenitally corrected transposition of great arteries
TV: Tricuspid valve
EF: Ejection fraction
NYHA: New York heart association
2D-TTE: Two dimentional transthoracic echocardiography
RT-3D-TEE: Real-time three dimentional transesophageal echocardiography
